# Modeling Age-Specific Mortality for Countries with Generalized HIV Epidemics

**DOI:** 10.1371/journal.pone.0096447

**Published:** 2014-05-22

**Authors:** David J. Sharrow, Samuel J. Clark, Adrian E. Raftery

**Affiliations:** 1 Department of Sociology, University of Washington, Seattle, Washington, United States of America; 2 Institute of Behavioral Science (IBS), University of Colorado at Boulder, Boulder, Colorado, United States of America; 3 MRC/Wits Rural Public Health and Health Transitions Research Unit (Agincourt), School of Public Health, Faculty of Health Sciences, University of the Witwatersrand, Johannesburg, South Africa; 4 Department of Statistics, University of Washington, Seattle, Washington, United States of America; 5 ALPHA Network, London School of Hygiene and Tropical Medicine, London, United Kingdom; 6 INDEPTH Network, Accra, Ghana; University of California, United States of America

## Abstract

**Background:**

In a given population the age pattern of mortality is an important determinant of total number of deaths, age structure, and through effects on age structure, the number of births and thereby growth. Good mortality models exist for most populations except those experiencing generalized HIV epidemics and some developing country populations. The large number of deaths concentrated at very young and adult ages in HIV-affected populations produce a unique ‘humped’ age pattern of mortality that is not reproduced by any existing mortality models. Both burden of disease reporting and population projection methods require age-specific mortality rates to estimate numbers of deaths and produce plausible age structures. For countries with generalized HIV epidemics these estimates should take into account the future trajectory of HIV prevalence and its effects on age-specific mortality. In this paper we present a parsimonious model of age-specific mortality for countries with generalized HIV/AIDS epidemics.

**Methods and Findings:**

The model represents a vector of age-specific mortality rates as the weighted sum of three independent age-varying components. We derive the age-varying components from a Singular Value Decomposition of the matrix of age-specific mortality rate schedules. The weights are modeled as a function of HIV prevalence and one of three possible sets of inputs: life expectancy at birth, a measure of child mortality, or child mortality with a measure of adult mortality. We calibrate the model with 320 five-year life tables for each sex from the World Population Prospects 2010 revision that come from the 40 countries of the world that have and are experiencing a generalized HIV epidemic. Cross validation shows that the model is able to outperform several existing model life table systems.

**Conclusions:**

We present a flexible, parsimonious model of age-specific mortality for countries with generalized HIV epidemics. Combined with the outputs of existing epidemiological and demographic models, this model makes it possible to project future age-specific mortality profiles and number of deaths for countries with generalized HIV epidemics.

## Introduction

The age pattern of mortality is a reflection of the age-specific underlying killing mechanisms and epidemiological profile of a population. Numerous mortality indicators like the under five mortality rate (the probability of death between birth and age 5, referred to as U5MR or 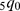) or the adult mortality rate (the probability that a 15 year old will die before reaching age 60 or 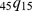) that are regularly used to track population health and development goals are often calculated from a complete set of age-specific period mortality rates. Moreover, for many countries without adequate vital registration systems, the total number of deaths in a population (the so-called ‘envelope’) used to allocate deaths by cause in global health reporting, such as the global burden of disease studies [1], [2], is calculated using model life tables to estimate all-cause mortality, and hence the total number of deaths. Accurate estimation of these age patterns is an essential step toward monitoring population wellbeing and working toward targeted public health actions.

Unfortunately, less than half the world’s countries have civil vital registration systems that function well enough to accurately count people and deaths by sex and age and thereby produce reliable measures of sex-age-specific mortality [3], [4]. This issue is particularly relevant to most countries experiencing high HIV prevalence. In the absence of this information, analysts use model life tables or other model-based approaches to estimate complete sets of sex-age-specific mortality rates based on a small number of mortality indictors such as life expectancy at birth or the U5MR. None of the existing model life tables are suitable for estimating mortality patterns for high HIV prevalence countries because they were not designed to reproduce the unique age pattern of deaths associated with HIV. HIV predominantly infects infants (from their mother) and young/middle-aged adults. Combined with the characteristic survival times for HIV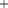 children and adults, this age pattern of incidence results in deaths concentrated in very young children and middle-aged adults. The result for the age profile of mortality is a spike in child mortality and a ‘hump’ in adult mortality located roughly in the middle of life [5]–[10].

**Figure 1:**
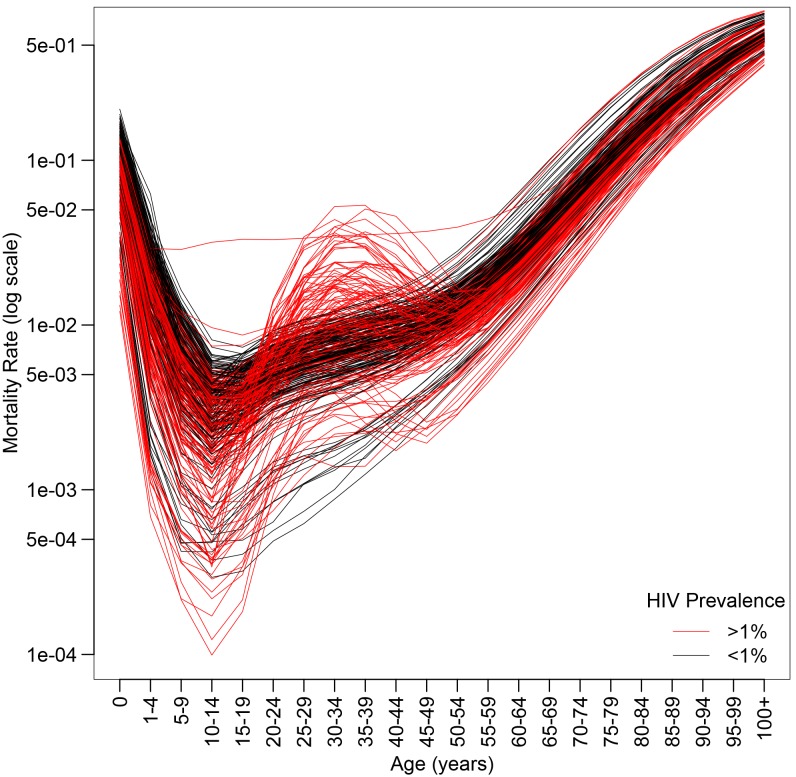
WPP 2010 female five-year mortality rate schedules for countries with generalized HIV epidemics 1970–2010. 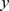
–axis on log scale. Country-periods with a generalized epidemic (>1% HIV prevalence) plotted in red.

**Figure 2:**
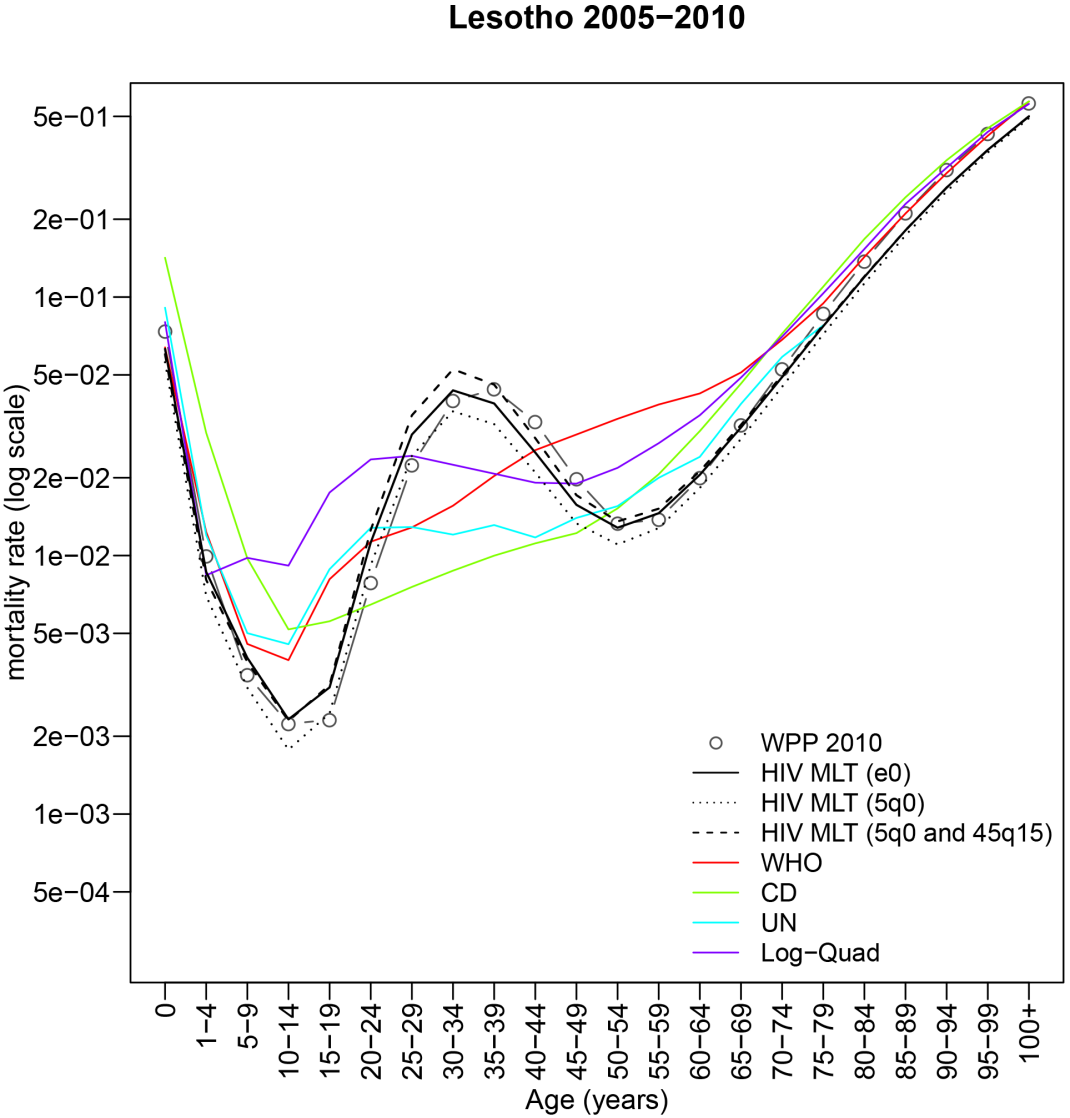
Fits of HIV MLT model with three different input combinations for Lesotho females 2005–2010. 1) HIV prevalence with life expectancy at birth [solid black line] 2) HIV prevalence and child mortality [dotted black line] 3) HIV prevalence with child mortality and adult mortality [dashed black line]. For comparison, fits from the WHO modified logit model [red solid line], Coale and Demeny model life tables [green solid line], UN model life tables for developing countries [teal solid line], and the Log-Quad model [purple solid line] are also shown.

The impact of endemic HIV on the age pattern of mortality is illustrated in Figure 1, which plots the five-year female mortality rate schedules from 1970–2010 for the 40 countries of the world experiencing a generalized HIV epidemic [11]. The mortality rate schedules produced under a generalized epidemic are plotted in red. A “generalized” HIV epidemic is defined by HIV prevalence greater than 1% in the general population and no concentration of the epidemic in high-risk subgroups such as men who have sex with men, intravenous drug users, or sex workers and their clients (From [12], p. 523): “Large increases in mortality associated with HIV/AIDS are observed in countries where prevalence in the general population is greater than about 1%. A stationary population with an expectation of life of 10 years, similar to the HIV-positive population, has a crude death rate of 1/10 = 0.1 or 100 per 1,000. A population containing 1% HIV-positive people will therefore add about 1 additional death per 1,000 to the crude death rate. Consequently, for populations with baseline (non-HIV) crude death rates of 10 per 1,000, an HIV prevalence of 1% corresponds to a significant 10% increase in the overall crude death rate.” Figure 1 clearly shows the accentuated adult mortality hump characteristic of generalized epidemics. Existing models of age-specific mortality and model life table systems have considerable trouble replicating the mortality profile generated under high HIV prevalence [13], [14]. Figure 2 plots the age-specific mortality rates for Lesotho females 2005–2010 (HIV prevalence 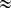
24% during this period and the previous five year period in Lesotho) along with the fits from four existing models: Coale and Demeny regional model life tables [15], [16], UN model life tables for developing countries [17], WHO modified logit model [18], and a recent and in many instances, remarkably accurate log-quadratic model based on data from the Human Mortality Database advanced by Wilmoth and colleagues [14], referred to as ‘Log-Quad’ in the remainder of this paper. Instead of fitting the hump itself, these models tend to produce a high, flat pattern of mortality that often matches the overall level of mortality, as measured by life expectancy at birth, but misses the actual age-specific rates. These fits do not reflect methodological shortcomings of any of these models but rather show that despite being the most widely used and accurate models of this type, they were not calibrated with data from high HIV prevalence countries/periods and are thus not designed to fit this type of pattern.

Model life tables also play a critical role in population projections. The United Nations Population Division (UNPD) produces population projections broken down by sex and age for all countries of the world every two years in the *World Population Prospects*. Although several organizations produce population projections, the UN projections have become the *de facto* standard [19], [20] and are used by international organizations and many governments whose statistical offices do not regularly produce such estimates and projections, a group that includes some of the high HIV prevalence countries. The UNPD’s current population projection methodology requires that age-specific mortality, fertility, and international migration rates be projected for each five-year period in the future. To obtain future mortality rates for countries not experiencing endemic HIV, the five-year gains in life expectancy at birth, 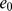, are projected [4] and then converted to age-specific rates based on model mortality patterns such as those contained in existing model life table systems. Age-specific mortality rates, especially during the course of an HIV epidemic, play a key role in future population size and age composition. Thus, the mortality component of projections for countries with generalized epidemics should include the influence of the future trajectory of the HIV epidemic and be able to produce the heavy adult mortality resulting from HIV/AIDS-related deaths.

### Aim

We present an HIV prevalence-calibrated model of sex-age-specific mortality that produces a complete set of sex-age-specific mortality rates as a function of HIV prevalence and one of three possible sets of additional inputs: life expectancy at birth, child mortality alone, or child mortality and adult mortality.

This objective and many of its detailed requirements are shaped by our ongoing collaboration with the UNPD to develop methods for probabilistic population estimation and projection [4], [20]–[22]. Consequently this HIV-calibrated model life table system is specifically designed to work well within the UNPD’s evolving population projection method. Current epidemiological and demographic models [23], [24] can make probabilistic projections of the key inputs. The mortality model presented here makes it possible to convert those inputs into probabilistic projections of age-specific mortality rates for countries with generalized HIV epidemics.

## Methods

### Data

The empirical base needed for this work is a comprehensive set of sex-age-specific mortality rates from populations with varying HIV prevalence that also span a wide range of HIV epidemic ages – i.e. ‘new’ to ‘old’ epidemics. While the need for variation in prevalence is obvious, the need for different epidemic ages arises from the changing dynamics of an epidemic as it ages and the fact that the characteristic age pattern of HIV incidence, prevalence, and mortality change as an epidemic ages and experiences interventions such as widespread use of antiretroviral therapy (ART). *Sufficient empirical data fitting these requirements do not exist* in a vetted, corrected, pooled, organized, documented fashion that can be used for a project such as this. The UNPD and many other potential users urgently need a new mortality model for populations with high HIV prevalence. Given these important needs, we have adopted a compromise solution that should work well enough for most purposes and is certainly better than having no HIV-calibrated model life table system. Our compromise is to use the set of life tables produced by the UNPD for countries with high HIV prevalence as part of their 2010 revision of the World Population Prospects [25]. The variation in age patterns of mortality in this collection of life tables is a systematic representation of what is known about HIV and mortality in populations with generalized epidemics. Furthermore, although not strictly empirical, this collection of life tables is *empirically-based* and uses most of the information we have on the effects of HIV on mortality by sex, age, HIV prevalence, and HIV epidemic stage (or age). We say ‘*most* of the information’ as the UNPD collection does not make systematic use of empirical data from demographic surveillance systems (DSS) such as those in the INDEPTH network (http://www.indepth-network.org/), HIV surveillance systems such as those from the ALPHA network (http://www.lshtm.ac.uk/eph/dph/research/alpha/), or certain sub-national surveys. Likewise, we refer to these data as ‘empirically-based’ in the sense that they are constructed from our best estimates of HIV prevalence and mortality and constrained to be consistent with long-term changes in population age structure, fertility, and mortality. These data are still not truly *empirical*, but they are the best we can do without embarking on a major data acquisition project. The structure and mechanics of the model we describe below do not depend on the data used to calibrate the model, so when a better, more empirical data set is available, it will be a straightforward task to recalibrate the model using the new data.

Resulting from the current unavailability of truly empirical data, we calibrate our model using a set of 320 five-year life tables for each sex from the World Population Prospects 2010 revision [25]. This dataset represents the 40 countries of the world experiencing a generalized HIV epidemic with eight five-year life tables from 1970–2010 for each country.

From the life tables we model the age-specific mortality rates (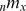 or the mortality rate from age 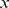 to 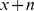) and derive the life expectancy at birth, child mortality (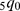 or the probability a newborn will die before his or her fifth birthday), and adult mortality (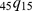 or the probability that a 15 year old will die before reaching his or her 60th birthday). The female log age-specific mortality rates are plotted for each country and time period in Figure 1. All life tables have uniform five-year age intervals except for the youngest age groups, up to an open interval of 100+ (0, 1–4, 5–9, 10–14,…, 100+). Finally, we obtain the mid-period (1973, 1978, 1983,…, 2008) HIV prevalence for adults age 15–49 as well as adult and child antiretroviral therapy (ART) coverage for each of the 320 country-periods from UNPD estimates used to produce WPP 2010 [26].

### Model

Our objective is a parsimonious model that can represent the age pattern of mortality rates for countries with generalized HIV epidemics as a function of HIV prevalence and some other mortality indicator (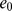, 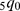 alone, or 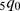 with 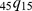). To accomplish this objective, we extend the component model of mortality that we developed earlier [27], [28] to include covariates, in this case HIV prevalence and various mortality indicators. As in [27] the general form of the model represents a set of age-specific mortality rates as the weighted sum of three independent, age-varying components that represent the age-varying nature of the mortality schedule:
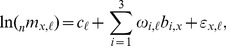
(1)where 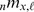 is the period age-specific mortality rate from age 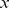 to age 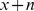 for life table 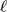, 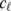 is a constant specific to life table 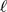, 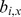 is the value of the 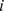 th component for age 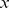, 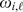 is the weight of the 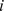 th component for life table 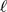, and 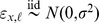 is the error term. The age-varying components 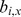 are fixed. Thus, the effective parameters in this model are the component weights 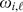, which are modeled as a function of HIV prevalence and an overall mortality indicator.

**Figure 3:**
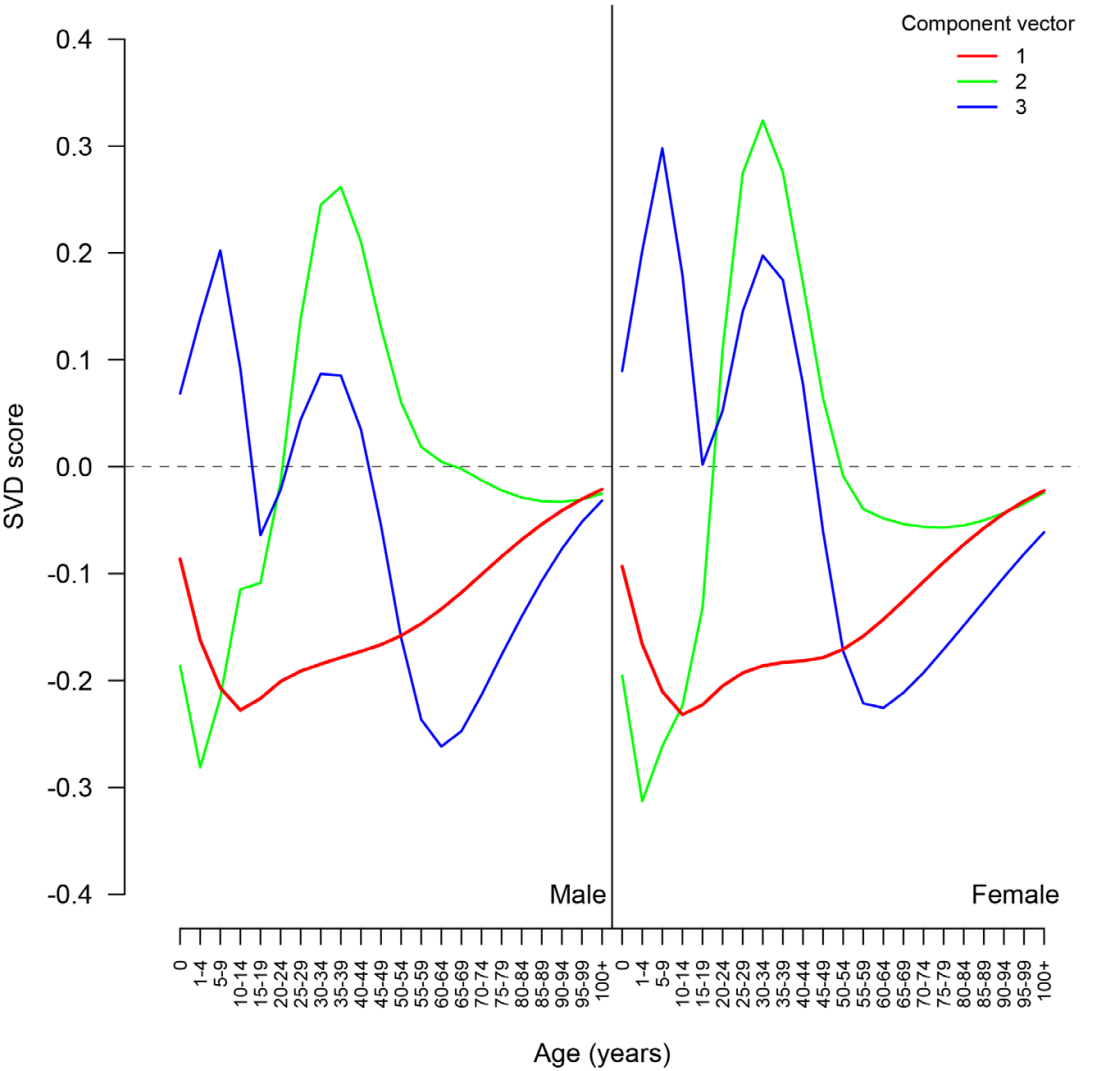
First three components (left-singular vectors). Derived from the Singular Value Decomposition of the World Population Prospects five-year mortality rate schedules 1970–2010 for countries with generalized HIV epidemics. 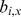 from Equation 1.

We first derive the age-varying components 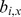 from a Singular Value Decomposition (SVD) of the matrix of observed mortality rate schedules. SVD decomposes a matrix into three smaller matrices including one whose columns are orthogonal and point in the directions with most variation in the original (22-dimensional) space – the *left singular vectors* (LSVs). The LSVs plotted in Figure 3 (and presented in Table S1) are the independent components we need, and they have the convenient property of encoding the bulk of the variation among the observed mortality schedules in a small number of vectors. We performed an in-sample validation similar to that outlined in the ‘Model Validation’ section using the model with a varying number of components and found little improvement in fit when including more than three components. The lack of improvement with inclusion of higher order components reflects the fact that each successive LSV accounts for a successively smaller proportion of the overall variance; the first three LSVs account for roughly 99.6% of the total age variation.

Following Clark [27], [28] we next regress each of the 320 mortality rate schedules for each sex on the first three left-singular vectors from the SVD yielding a set of weights 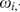, ordinary least squares regression coefficients, for each country-period life table. These weights relate the individual mortality rate schedule to the SVD components. Again using ordinary least squares, each 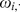 is modeled as a linear combination of HIV prevalence and one of three sets of mortality indictors: life expectancy at birth, child mortality alone, or child mortality and adult mortality. We use Bayesian model selection [29] to find the combination of these variables that best explains each 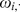 (this step was performed using the BMA package [30] in the statistical analysis software R). In model selection we included ART coverage for both adults and children among the set of potential predictor variables along with HIV prevalence and the mortality indicator, but neither adult nor child ART coverage was found to predict the weights.

We fit separate models for each 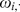 by sex as well as for African countries and non-African countries (Bahamas, Belize, Guyana, Haiti, and Jamaica). The region-sex-specific model coefficients for each 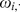 are given in Tables S2, S3, and S4. Using the coefficients presented in these tables, for a given HIV prevalence and value(s) of one of the three mortality input combinations, one can produce predicted values of 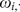, denoted by 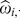, which when substituted into Equation 1 produce a set of predicted mortality rates.

Finally, we adjust the predicted mortality rates so that the mortality indicators output from the model match the input values. When the input indicator is life expectancy at birth, we adjust 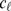 in Equation 1 in such a way that the output mortality rates produce a life expectancy that matches the input life expectancy. This adjustment typically alters the level of mortality rather than its shape because the pattern of predicted mortality rates is largely determined by the 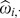
’s. When the input indicator is child mortality alone, we raise the first two mortality rates, 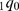 and 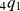, to the power 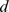, where 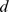 is the solution to the equation:
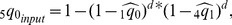
(2)namely 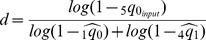. When child mortality and adult mortality are used as the inputs, we use a combination of the two adjustments just described. We adjust 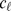 to match 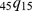 as we did with 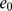 and then match 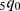 using Equation 2. In practice, the predicted rates typically need very little adjustment, but this approach ensures that the output mortality indicators match the inputs.

### Our Model in Relation to Existing Mortality Models

We now describe how our model compares to existing mortality models that also use matrix factorization to generate reference age patterns.

The SVD is a general linear algebra technique used to factor an arbitrary rectangular matrix 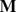 into three new matrices related to each other by the product 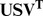. Intuitively, imagine the column vectors of 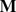 as a cloud of points; the SVD identifies a new orthonormal basis (the right singular vectors 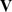) for this cloud. The singular values (diagonal matrix 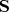) indicate the characteristic magnitude of the cloud in the direction of each new dimension. The product of the singular values and the left singular vectors (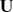) locate each point along the new dimensions. SVD does not require the cloud to be centered (mean-subtracted), and consequently, in general the new basis identified by SVD does not line up with the axes of maximum variation in the cloud.

Principal Components Analysis (PCA) is a related technique that is equivalent to a specific application of SVD. PCA aims to identify the axes of maximum variation in a cloud of data points. It does this by finding the eigen decomposition of the covariance (or correlation) matrix associated with the data. The covariance matrix is a description of a *centered* version of the data and also square; eigen decomposition requires a well-behaved square matrix. PCA is equivalent to an SVD on a centered version of the data; in this specific circumstance, the eigenvectors and right singular vectors identify the same orthogonal axes along which there is maximum variation in the cloud of data points, and the square roots of the eigenvalues are equal to the singular values.

Several mortality models use factorization methods like SVD and PCA to represent aspects of the age pattern of mortality. The Lee-Carter model [31] uses an SVD factorization of mean-substracted, logged age-specific mortality rates to provide the age-specific components of the model (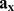 and 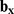 in Section 3, p 661 of [31]) and the Log-Quad model [14] uses an SVD factorization to extract the characteristic age-pattern (the first LSV) from the residuals produced from fitting the model without the ‘
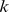
’ correction component (Equation A6, p 28 of [14]). This age pattern of residuals is then scaled using the ‘
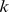
’ correction parameter in the model. The UN model life tables for developing countries [17] also use PCA to depict the age pattern of mortality change within regional clusters.

Although our model also uses an SVD factorization, the structure of our model is different from other mortality models, including the three just mentioned, and the SVD-derived components are both different and incorporated in a new way. Our model does not have families like the UN model life tables, does not use SVD-like components to model residuals like either the Log-Quad or UN model life tables, and does not incorporate a particular functional form like the Log-Quad model. Our model is most similar to the Lee-Carter model but differs in important ways. The Lee-Carter model uses the SVD to factorize the mean-subtracted log mortality rates (like a PCA) into a component that represents the basic age pattern of mortality and a second component that represents time-dependent, age-specific changes in mortality. Unlike all the others discussed here, including ours, the Log-Quad model exploits the general curvilinear relationship between child mortality and mortality at older ages and builds this relationship into the model so that non-child mortality is a quadratic function of child mortality. The SVD-derived ‘
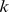
’ correction component is used to ensure that the age pattern of non-child mortality is reasonable (and flexible); essentially it implements an age-specific fine-tuning of the fundamental prediction of non-child mortality from child mortality.

Our model is simpler in its general form and designed to be able to incorporate any collection of possible predictors. We use the SVD to factorize observed log mortality (without subtracting the mean, i.e. unlike PCA) into 1) a basic age pattern of log mortality and 2) 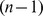 additional orthogonal components that represent age patterns of deviation from the basic pattern, where 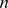 is the number of life tables in the dataset. These factors are the LSVs, and we find that three are sufficient to reproduce the important age-based variation in the data. Any of the observed age patterns of mortality in the original dataset can be reproduced to within desired precision by taking a weighted sum of enough components. Unlike the other models described, we further model the weights of the three components that we keep as linear functions of HIV prevalence and various indicators of mortality level (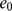, 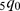 and/or 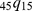). Operating through the weights, these parameters adjust both level and shape simultaneously to produce a continuously varying series of life tables. So although we use some of the same ingredients, our model is conceptually and operationally distinct from existing mortality models.

## Results

### Calibration Data

HIV prevalence for the African life tables ranges from 0 to 26% with a mean of 3.7%, while for the non-African life tables prevalence is much lower overall, ranging from 0 to 3.8% with a mean of 1.3%. Likewise, life expectancy among the African life tables is considerably lower than for the five non-African countries. African male life expectancy ranges from approximately 22 to 62 years with a mean of 48.3, and African female life expectancy ranges from 25.6 to 65.8 with a mean of 51.1. Male life expectancy from the non-African countries ranges from 46.7 to 74 with a mean of 64, and female non-African life expectancy ranges from 49.3 to 77.8. These differences in the distributions of the input parameters are what drives the need for region-sex-specific models and are reflected in the region-specific distribution of weights 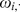. A summary of the 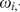 values for African and non-African life tables is shown in Table 1. Note the overall lower distribution of the first weight 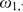 for African countries. When multiplied by the first LSV (red line plotted in Figure 3), which has negative values at all ages, the smaller African weights produce a higher overall level of mortality, which is then altered in a life expectancy-constant way to reflect the influence of HIV prevalence on the age pattern of mortality.

**Table 1:** Summary of the distribution of 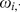 values for African (n = 260) and non-African life tables (n = 40).

	Africa			Non-Africa		
	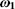	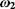	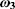	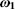	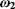	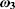
Minimum	18.73	−1.47	−2.08	24.89	−0.72	−2.69
Median	25.24	−0.58	0.47	31.73	0.51	−0.94
Mean	25.36	−0.17	0.21	31.07	0.83	−1.09
Maximum	30.01	4.27	1.91	35.72	3.28	−0.15

### Model Output

The output from this model is a complete set of predicted log sex-age-specific mortality rates that reflect HIV prevalence and can be used to calculate a full life table from which various mortality indictors can be generated. The effective parameters are the weights 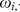 derived from region- and sex-specific models. The model will produce a full set of sex-age-specific mortality rates for any combination of HIV prevalence and mortality indicator – life expectancy at birth, child mortality alone or child mortality with adult mortality.

**Figure 4:**
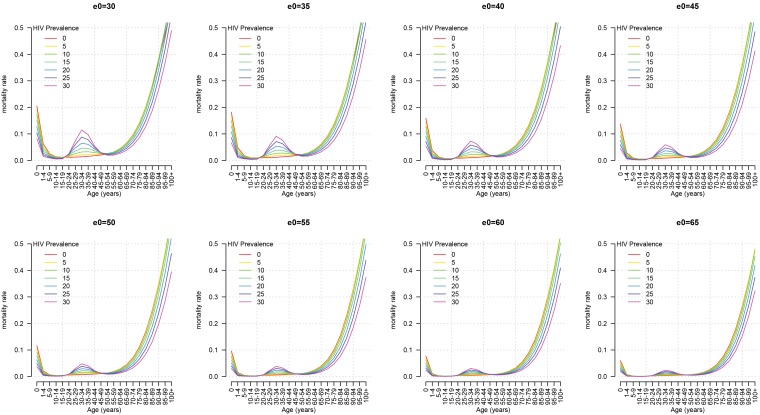
Model output for HIV MLT female Africa model at varying prevalence and varying 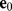.

**Figure 5:**
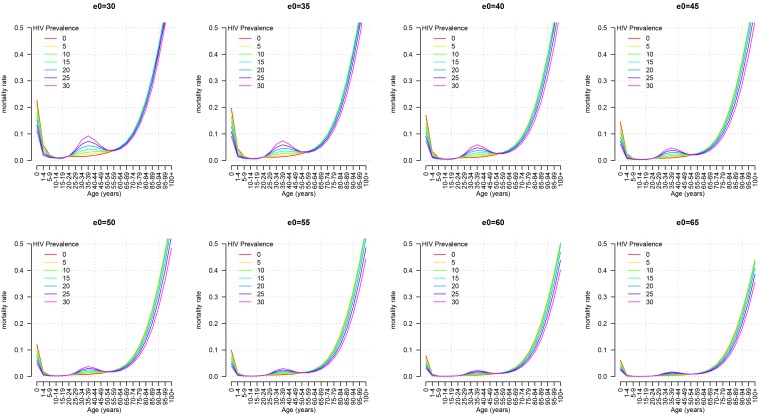
Model output for HIV MLT male Africa model at varying prevalence and varying 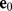.

Model outputs for various combinations of HIV prevalence and life expectancy for Africa are presented in Figures 4 and 5 for females and males respectively. Figures 4 and 5 illustrate the range of patterns our model can produce and reveal another desirable property of this model. ART coverage is not related to (does not predict) the weights 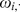, but the effect of ART on adult mortality is not ignored. Once ART coverage reaches near universal levels, a paradox emerges in which both life expectancy and HIV prevalence *increase together* as seropositive individuals live longer in the population and the HIV mortality hump is pushed to older and older ages, eventually merging with the natural increase in mortality at advanced ages [32]–[34]. The model must be able to represent this relationship in the future. Figures 4 and 5 confirm that the model can do this. At higher levels of life expectancy, the effect of prevalence is mitigated and the adult mortality hump nearly disappears, even at very high prevalence.

### Model Validation

In order to evaluate prediction accuracy and the robustness of our modeling strategy, we carried out a cross validation exercise where we calibrated the model (for all three input combinations) with a random 75% sample of the data, and then predicted the remaining 25%. We ascertained fit by calculating the mean absolute error (MAE) for the (non-logged) mortality rates among all age groups (0–75) and life tables in the prediction sample along with the MAE for life expectancy at birth 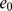, under-5 mortality 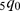, and adult mortality 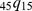. For comparison with existing models of all-age mortality, we predicted the 25% held-out sample using the WHO modified logit system, the UN model life tables for developing countries, the Coale-Demeny regional model life tables, and the Log-Quad model. The UN system was fit using the procedure described on page 2 of Chapter IV of [17], *Model Life Tables for Developing Countries*, where the complete set of age-specific probabilities of death is used to estimate the appropriate loading factor. To fit the Coale and Demeny life tables, we first selected the level by matching life expectancy to the closest half year and then selected the regional pattern that minimizes the sum of squared errors from the observed mortality rates. The WHO system was fit using the STATA software inputing 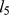, survivorship to age 5, and 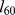, survivorship to age 60, to the “modmatch” function obtained from http://www.who.int/healthinfo/global_burden_disease/tools_software/en/. The Log-Quad model was fit with R code provided at http://www.demog.berkeley.edu/~jrw/LogQuad/ and takes 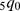 and 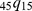 as inputs. Repeating this procedure 1,000 times yields a distribution of these fit metrics. The means of these distributions are presented in Table 2. Because the system described in this paper is essentially an HIV-calibrated model life table system we refer to it as ‘HIV MLT’. For all metrics, smaller numbers suggest a better overall fit and the smallest number in each column is bolded.

**Table 2:** Cross-validation results showing the mean of the distribution of mean absolute error (MAE) for three mortality indicators and amongst all-ages and life tables after 1,000 iterations of cross validation.

	Male				Female			
Model	All-ages 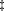	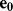	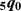	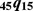	All-ages 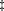	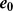	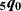	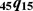
HIV MLT 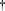								
input: 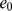	3.43	–	**13.05**	**21.81**	3.18	–	**12.26**	**22.82**
input: 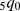	3.10	1.22	–	29.33	2.84	1.45	–	32.47
input: 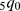 and 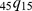	**2.80**	**0.56**	–	–	**2.80**	**0.57**	–	–
WHO 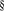	5.88	1.16	–	–	4.65	0.91	–	–
CD 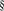	3.84	–	16.40	37.26	3.33	–	18.51	35.98
UN	6.32	1.13	13.92	36.85	4.98	1.51	28.84	38.65
Log-Quad 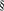	4.45	0.60	–	–	3.87	0.78	–	–

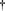
HIV MLT refers to the model presented in this paper with three possible input combinations: 1) HIV prevalence with life expectancy at birth [input: 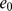], 2) HIV prevalence with child mortality [input: 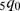], or 3) HIV prevalence with child mortality and adult mortality [input: 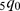 and 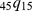]. MAE for ‘All-ages’, 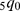, and 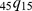 expressed per 1,000 and the smallest number in each column is bolded. For HIV MLT model, ‘–’ indicates MAE 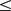
0.001 (or 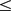
0.01 for 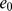).

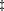
‘All-ages’ refers to the mean absolute error for the non-logged mortality rates across age groups (0, 1–4, 5–9, 10–14,…, 75) and amongst all life tables (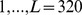).

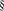
‘WHO’, ‘CD’, and ‘Log-Quad’ contain blank spaces as these quantities are inputs to these systems and thus have no error.

Results from Table 2 show that our model is able to outperform the other four existing model life table systems when fitting mortality data generated under high HIV prevalence. This result is no surprise because those models were not designed to replicate the age pattern of mortality in populations with generalized HIV epidemics. No matter which input combination is used, the HIV MLT model achieves a smaller MAE among all ages and country-periods (‘All-ages’ column) compared to the four other models. The HIV MLT model, with the input combination of HIV prevalence and 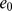, shows the second lowest ‘All-ages’ MAE, second only to the input combination of HIV prevalence, child mortality, and adult mortality, which uses slightly more information as inputs. The HIV MLT model shows mean absolute errors for life expectancy of less than one year for both sexes when using child and adult mortality along with HIV prevalence as inputs, and slightly more than one year when using child mortality alone and HIV prevalence. The Log-Quad model, arguably the most accurate of the four we use for comparison, shows small errors for life expectancy at birth of less than a year but misses the age-specific mortality rates (see Figure 2). Our model also shows modest errors in predicting the probability of childhood death when using 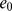 as an input; these errors are about 1.3 and 1.2 percentage points for the male and female models respectively. The error in predicting the probability of adult death is slightly higher than for childhood death at around 2 percentage points for both sexes.

The key advantage of this model is its ability to produce the accentuated adult mortality humps associated with a generalized HIV epidemic. Figure 2 plots the predicted 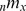 schedule from the HIV MLT model along with the WHO, Coale and Demeny, UN, and Log-Quad fits for Lesotho females in 2005–2010. Figure 2 makes clear that when HIV prevalence is high and the HIV hump is present, the HIV MLT model is able to reproduce age-specific mortality accurately. Additional selected fits can be found in Figures S1, S2, S3, and S4. The MAE by prevalence from an in-sample validation where we fit each of the 320 mortality schedules with the model described in this paper and the four comparison models is shown in Table S5 and Figure S5. This in-sample exercise shows that the HIV MLT model performs consistently at various prevalence levels and is able to outperform the four comparison models at very high HIV prevalence.

## Discussion

We have presented a flexible, parsimonious model of age-specific mortality for countries with generalized HIV epidemics. First, a set of age-specific mortality rates is represented as the weighted combination of a set of age-varying components. Next, the weights are modeled as a function of mortality indicators and HIV prevalence. This structure allows us to map HIV prevalence combined with other mortality indicator(s) onto a set of age-specific mortality rates reflecting the impact of a generalized HIV epidemic.

At present, our model does not include ART coverage as an input parameter as it was not a strong predictor of any of the weights. Although life expectancy at birth (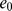) likely captures at least some of the relationship between ART coverage and age-specific mortality rates, as ART coverage becomes more widespread (thus increasing the variation in ART coverage across countries and time periods), capturing both effects with just 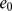 may become more problematic. Fortunately, the model structure is flexible enough to include other relevant, population-level covariates such as ART coverage or GDP if and when these quantities are available.

Results from a cross-validation experiment suggest that a three-component model fits best with modest errors for several mortality indicators. The cross validation results show that our model is able to predict age-specific mortality for countries with generalized HIV epidemics better than existing model life table systems. Combined with the outputs of existing epidemiological and demographic models, this model makes it possible to estimate future mortality profiles for countries with generalized HIV epidemics. The method also makes it possible to use other mortality indicators as well as social or economic variables to model the weights and hence predict age-specific mortality.

Because the comprehensive empirical data necessary to calibrate this model do not exist, we have been forced to compromise and use modeled mortality data produced by the UNPD. One consequence is that our model reproduces the variability in HIV prevalence-sex-age-specific mortality embodied in the UNPD life tables. Insofar as they are correctly related to the empirical ‘truth’ and cover the full range of possible HIV prevalence-sex-age-specific mortality, then our model does so too. Whatever variation is not included in the UNPD life tables is also not included in our model. This is an important limitation of this work, but we feel it is justified in light of the urgent and consequential need for a model of this type, especially for use by the UNPD. We are confident that the model is ‘fit for purpose’ in the context of the UNPD’s requirement to produce population projections for countries with high HIV prevalence. To address this limitation, the next phase of this work will involve creation of the empirical data set that we need, working in collaboration with the INDEPTH and ALPHA networks of health and demographic and HIV surveillance sites in Africa and Asia that do have the data that we need in raw form.

### R Package

We have released an R package, HIV.LifeTables [35], that implements the model described in this paper, and we will continue to develop and improve that package. The package is available as a standard R package from the Comprehensive R Archive Network (CRAN) that can be run using the R statistical software.

## Supporting Information

Figure S1 **Fits of HIV MLT model with three different input combinations to Swaziland female five-year life tables 1980–2010.** 1) HIV prevalence with life expectancy at birth [solid black line] 2) HIV prevalence and child mortality [dotted black line] 3) HIV prevalence with child mortality and adult mortality [dashed black line]. For comparison, fits from the WHO modified logit model [red solid line], Coale and Demeny model life tables [green solid line], UN model life tables for developing countries [teal solid line], and the Log-Quad model [purple solid line] are also shown.
(TIF)

Figure S2 **Fits of HIV MLT model with three different input combinations to Ghana female five-year life tables 1980–2010.** 1) HIV prevalence with life expectancy at birth [solid black line] 2) HIV prevalence and child mortality [dotted black line] 3) HIV prevalence with child mortality and adult mortality [dashed black line]. For comparison, fits from the WHO modified logit model [red solid line], Coale and Demeny model life tables [green solid line], UN model life tables for developing countries [teal solid line], and the Log-Quad model [purple solid line] are also shown.
(TIF)

Figure S3 **Fits of HIV MLT model with three different input combinations to Haiti male five-year life tables 1980–2010.** 1) HIV prevalence with life expectancy at birth [solid black line] 2) HIV prevalence and child mortality [dotted black line] 3) HIV prevalence with child mortality and adult mortality [dashed black line]. For comparison, fits from the WHO modified logit model [red solid line], Coale and Demeny model life tables [green solid line], UN model life tables for developing countries [teal solid line], and the Log-Quad model [purple solid line] are also shown.
(TIF)

Figure S4 **Fits of HIV MLT model with three different input combinations to South Africa male five-year life tables 1980–2010.** 1) HIV prevalence with life expectancy at birth [solid black line] 2) HIV prevalence and child mortality [dotted black line] 3) HIV prevalence with child mortality and adult mortality [dashed black line]. For comparison, fits from the WHO modified logit model [red solid line], Coale and Demeny model life tables [green solid line], UN model life tables for developing countries [teal solid line], and the Log-Quad model [purple solid line] are also shown.
(TIF)

Figure S5 **Mean Absolute Error for ages 0–75 for all model life table systems by sub-ranges of HIV prevalence.** Y-axis scaled to be per 1,000.
(TIF)

Table S1
**First three left singular vectors from the Singular Value Decomposition of the matrix of mortality rates from WPP 2010 for the 40 countries experiencing a generalized HIV epidemic.** 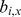 from Equation 1 and plotted in Figure 3.(PDF)

Table S2
**Coefficients for modeled weights 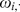 as a function of 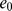 and prevalence.** Given values of 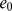 and prevalence, these models will produce weights 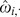 that when inserted into Equation 1 will produce a complete set of age-specific mortality rates.(PDF)

Table S3
**Coefficients for modeled weights 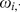 as a function of 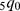 and prevalence.** Given values of 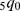 and prevalence, these models will produce weights 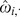 that when inserted into Equation 1 will produce a complete set of age-specific mortality rates.(PDF)

Table S4
**Coefficients for modeled weights 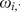 as a function of 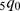, 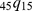, and prevalence.** Given values of 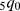, 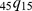, and prevalence, these models will produce predicted weights 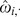 that when inserted into Equation 1 will produce a complete set of age-specific mortality rates.(PDF)

Table S5 **Mean Absolute Error for ages 0–75 for all model life table systems by sub-ranges of HIV prevalence.** HIV prevalence ranges are shown at the top of each column. All numbers in this table are per 1,000.
(PDF)
