# Supplementary figures and images for: Modeling Age-Specific Mortality for Countries with Generalized HIV Epidemics
Source: PLoS One. 2014 May 22;9(5):e96447. doi: 10.1371/journal.pone.0096447 (PMC4031074; doi:10.1371/journal.pone.0096447)

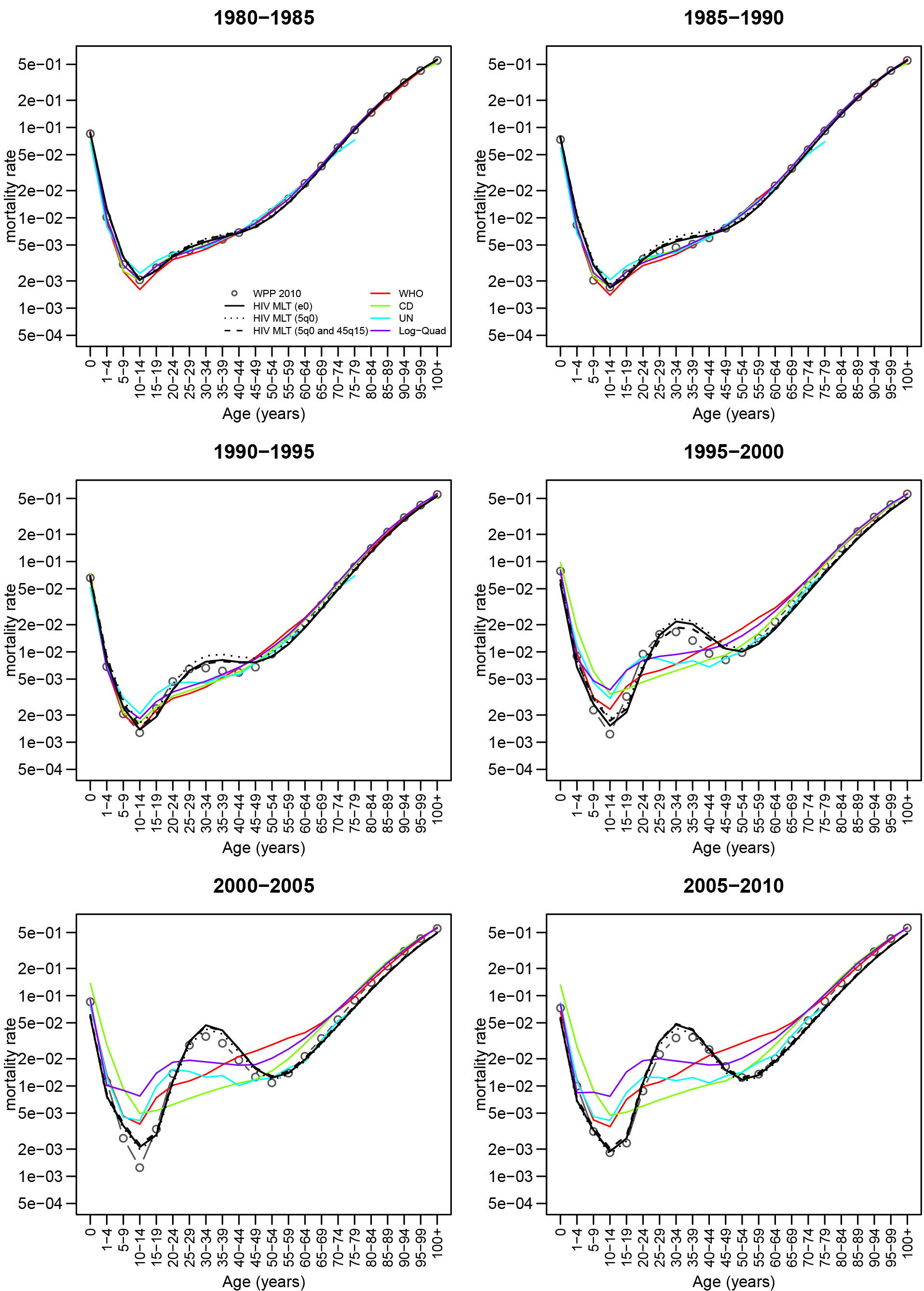

Supplement: Figure S1 — Fits of HIV MLT model with three different input combinations to Swaziland female five-year life tables 1980–2010. 1) HIV prevalence with life expectancy at birth [solid black line] 2) HIV prevalence and child mortality [dotted black line] 3) HIV prevalence with child mortality and adult mortality [dashed black line]. For comparison, fits from the WHO modified logit model [red solid line], Coale and Demeny model life tables [green solid line], UN model life tables for developing countries [teal solid line], and the Log-Quad model [purple solid line] are also shown. (TIF) [file pone.0096447.s001.tif]

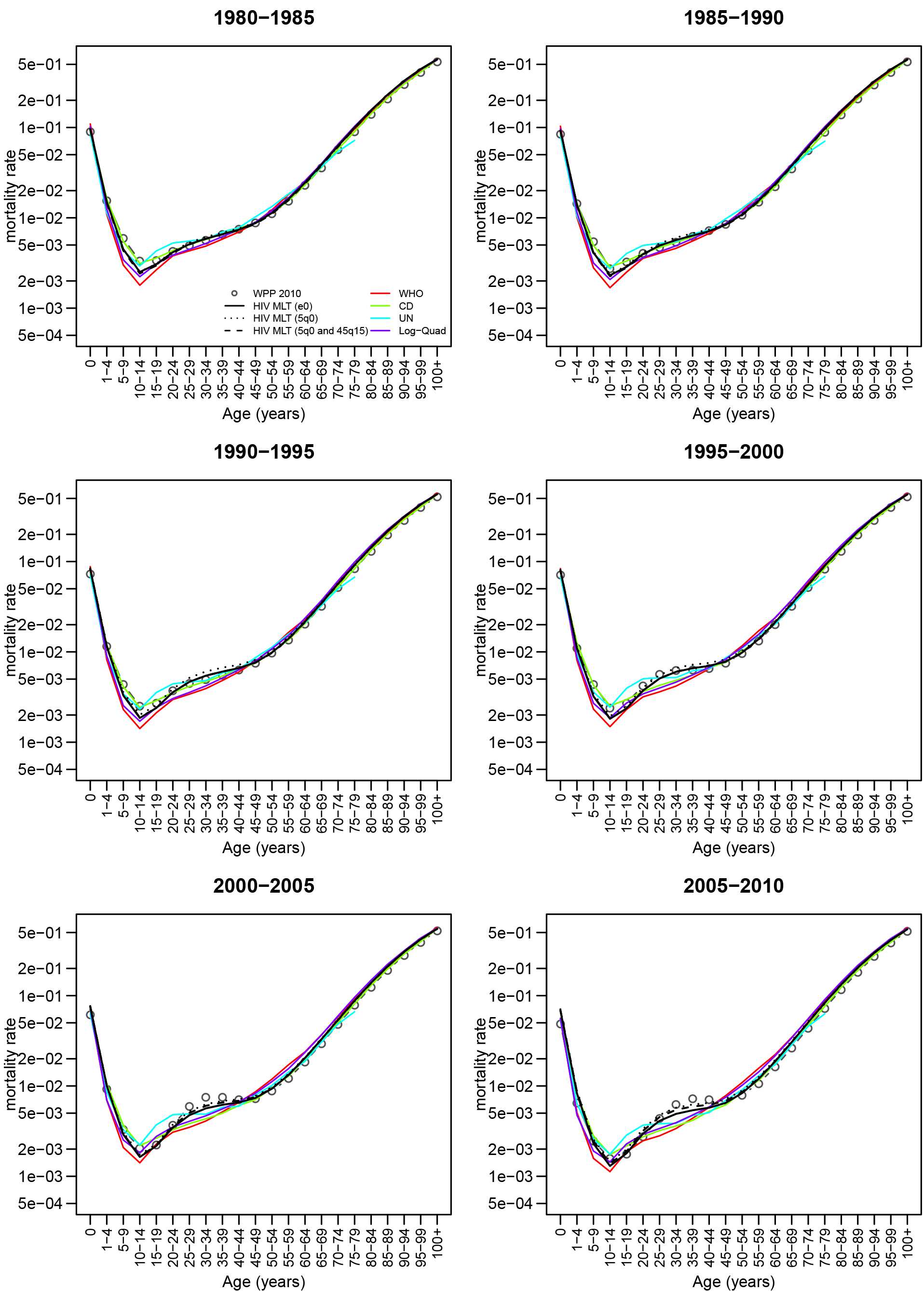

Supplement: Figure S2 — Fits of HIV MLT model with three different input combinations to Ghana female five-year life tables 1980–2010. 1) HIV prevalence with life expectancy at birth [solid black line] 2) HIV prevalence and child mortality [dotted black line] 3) HIV prevalence with child mortality and adult mortality [dashed black line]. For comparison, fits from the WHO modified logit model [red solid line], Coale and Demeny model life tables [green solid line], UN model life tables for developing countries [teal solid line], and the Log-Quad model [purple solid line] are also shown. (TIF) [file pone.0096447.s002.tif]

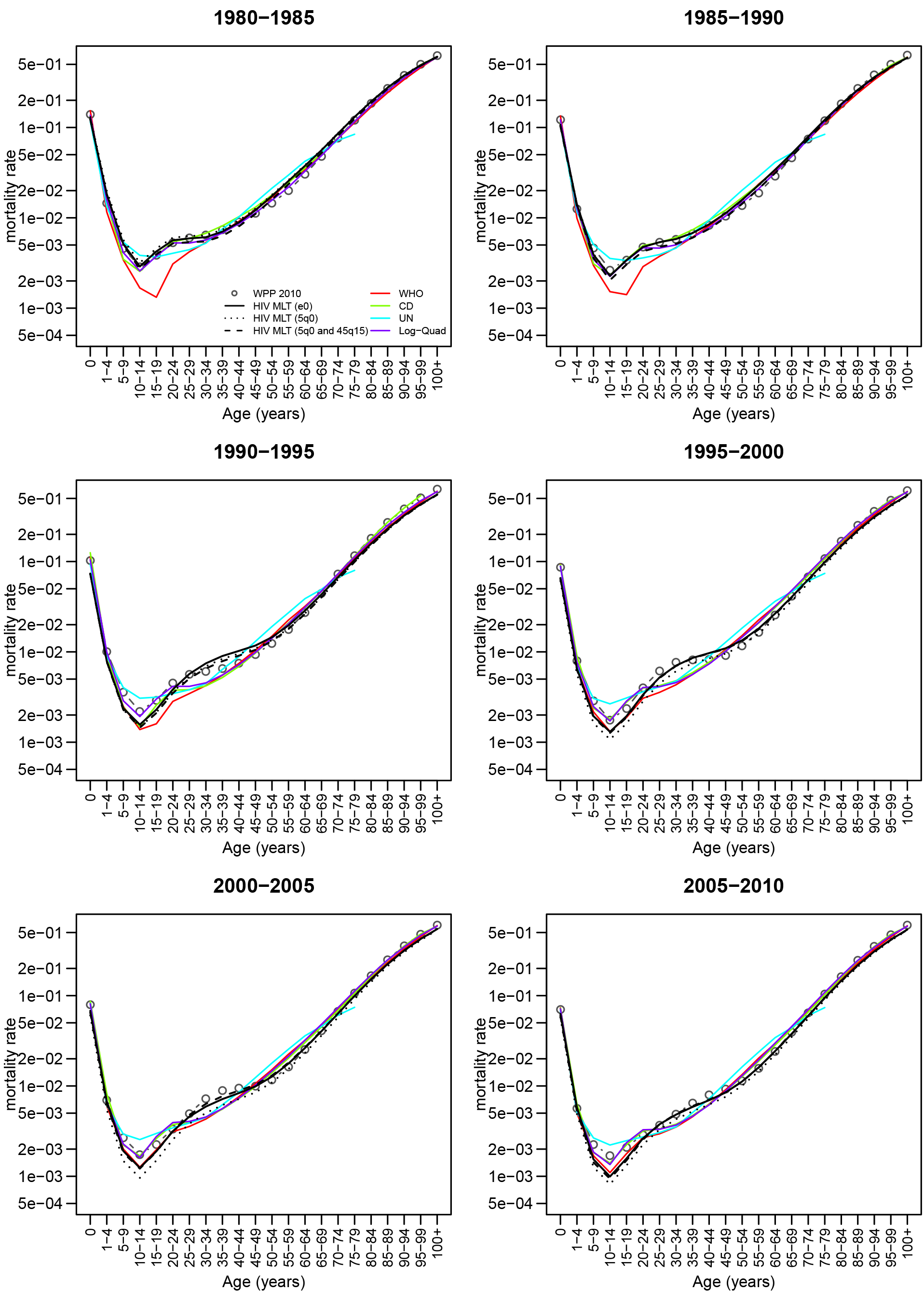

Supplement: Figure S3 — Fits of HIV MLT model with three different input combinations to Haiti male five-year life tables 1980–2010. 1) HIV prevalence with life expectancy at birth [solid black line] 2) HIV prevalence and child mortality [dotted black line] 3) HIV prevalence with child mortality and adult mortality [dashed black line]. For comparison, fits from the WHO modified logit model [red solid line], Coale and Demeny model life tables [green solid line], UN model life tables for developing countries [teal solid line], and the Log-Quad model [purple solid line] are also shown. (TIF) [file pone.0096447.s003.tif]

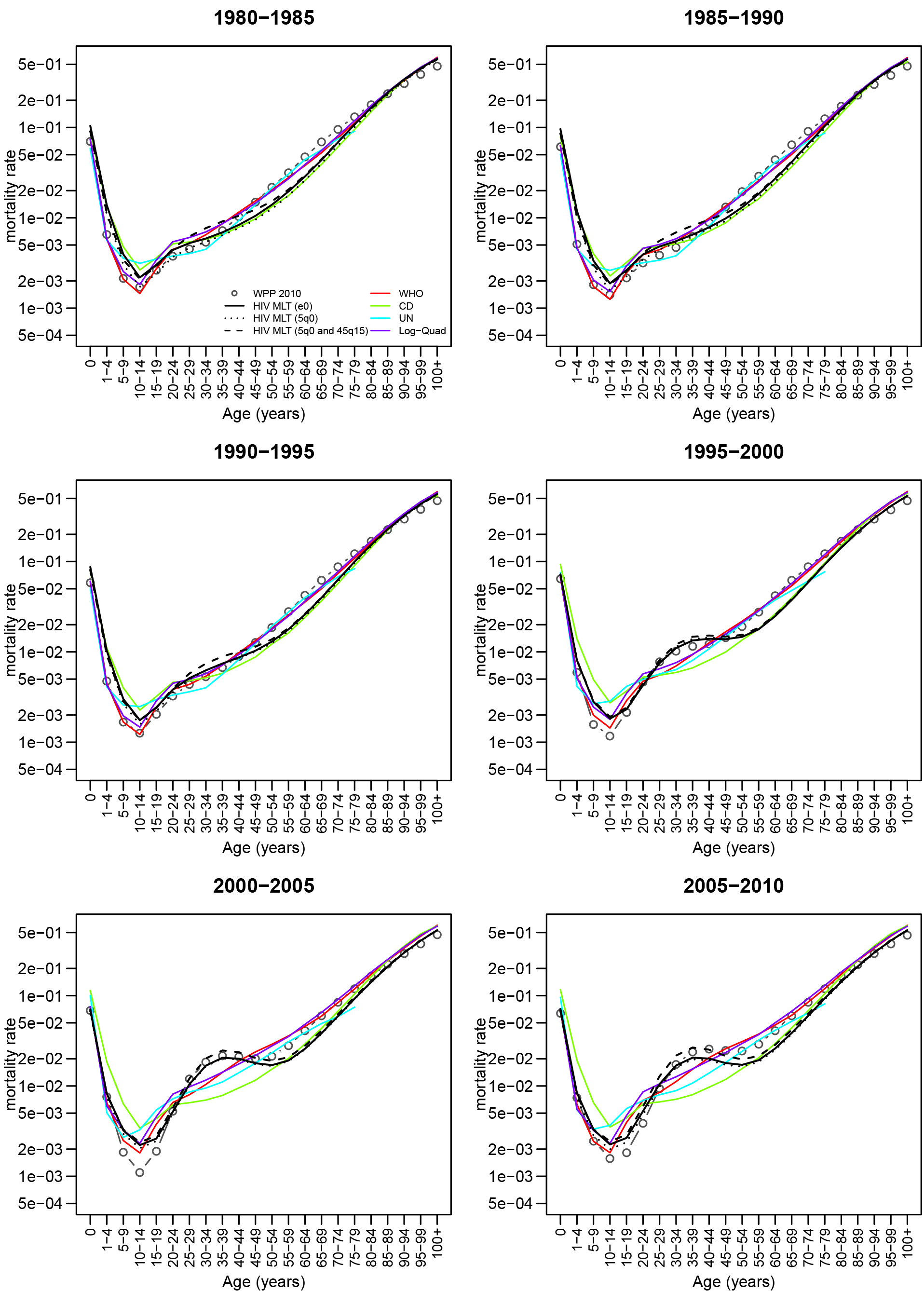

Supplement: Figure S4 — Fits of HIV MLT model with three different input combinations to South Africa male five-year life tables 1980–2010. 1) HIV prevalence with life expectancy at birth [solid black line] 2) HIV prevalence and child mortality [dotted black line] 3) HIV prevalence with child mortality and adult mortality [dashed black line]. For comparison, fits from the WHO modified logit model [red solid line], Coale and Demeny model life tables [green solid line], UN model life tables for developing countries [teal solid line], and the Log-Quad model [purple solid line] are also shown. (TIF) [file pone.0096447.s004.tif]

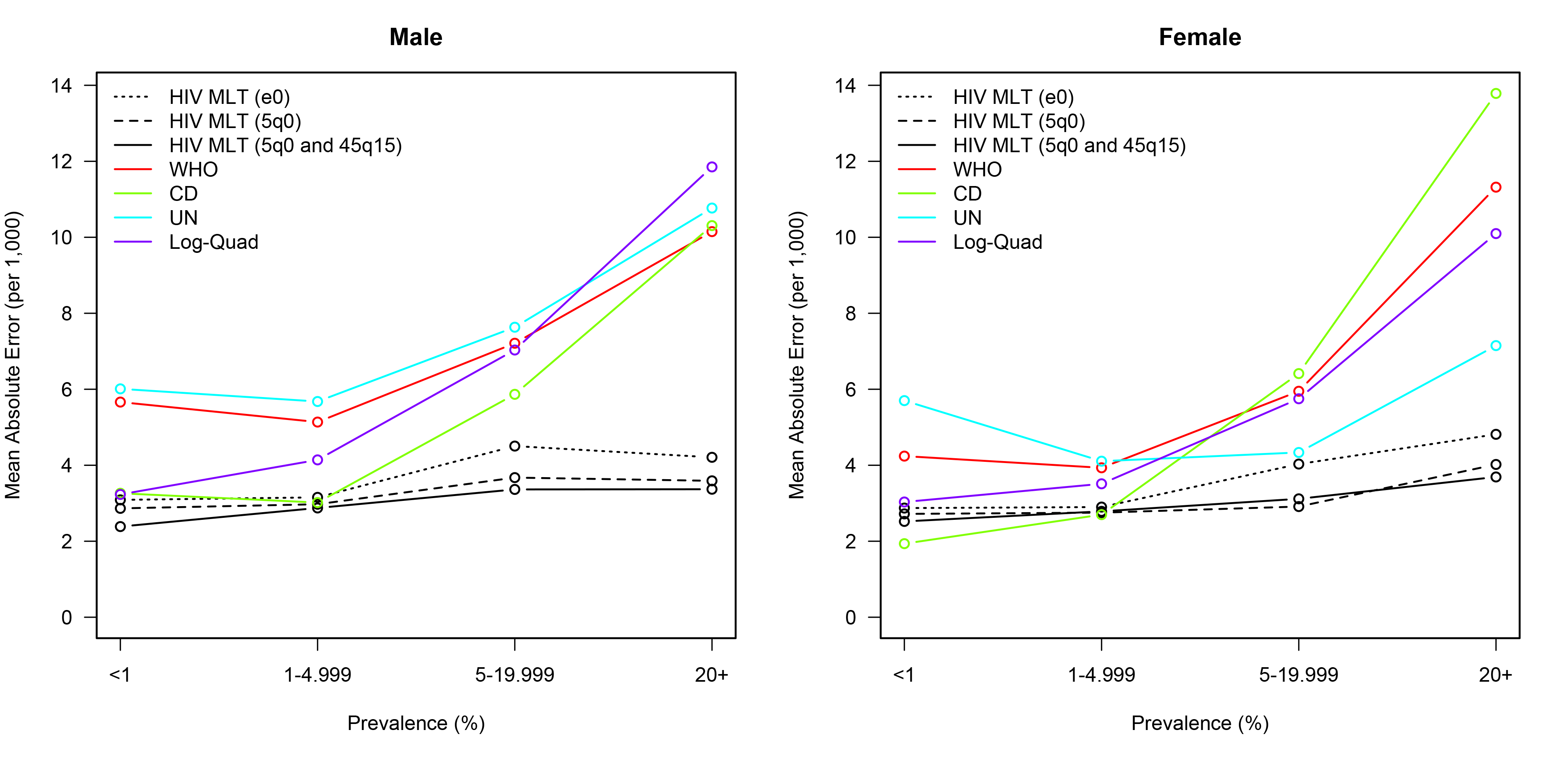

Supplement: Figure S5 — Mean Absolute Error for ages 0–75 for all model life table systems by sub-ranges of HIV prevalence. Y-axis scaled to be per 1,000. (TIF) [file pone.0096447.s005.tif]
