# Supplementary material for: Modeling Age-Specific Mortality for Countries with Generalized HIV Epidemics
Source: PLoS One. 2014 May 22;9(5):e96447. doi: 10.1371/journal.pone.0096447 (PMC4031074; doi:10.1371/journal.pone.0096447)
Supplement: Table S1 — First three left singular vectors from the Singular Value Decomposition of the matrix of mortality rates from WPP 2010 for the 40 countries experiencing a generalized HIV epidemic. from Equation 1 and plotted in Figure 3. (PDF) [file pone.0096447.s006.pdf]

Modeling Age-Specific Mortality for Countries with Generalized HIV Epidemics: Table S1

Table S1. First three left singular vectors from the Singular Value Decomposition of the matrix of mortality rates from WPP 2010 for the 40 countries experiencing a generalized HIV epidemic. $b_{i,x}$ from Equation 1 and plotted in Figure 2.

	Male			Female		
Age (x)	$b_{1,x}$	$b_{2,x}$	$b_{3,x}$	$b_{1,x}$	$b_{2,x}$	$b_{3,x}$
0	-0.0863831	-0.1863456	0.0684241	-0.0933371	-0.1953952	0.0896329
1-4	-0.1622137	-0.2812324	0.1387730	-0.1660563	-0.3129614	0.2019198
5-9	-0.2068765	-0.2158233	0.2023899	-0.2103099	-0.2615687	0.2977576
10-14	-0.2276872	-0.1146871	0.0916813	-0.2318174	-0.2235830	0.1796178
15-19	-0.2167700	-0.1086496	-0.0641072	-0.2227304	-0.1320417	0.0019539
20-24	-0.2009026	-0.0144001	-0.0218221	-0.2050775	0.1097540	0.0523490
25-29	-0.1911840	0.1384402	0.0441272	-0.1929131	0.2745561	0.1452674
30-34	-0.1846376	0.2450371	0.0870204	-0.1862256	0.3239613	0.1975500
35-39	-0.1785384	0.2618508	0.0853558	-0.1831307	0.2754657	0.1746882
40-44	-0.1727718	0.2104490	0.0349015	-0.1816644	0.1717950	0.0773590
45-49	-0.1664254	0.1301240	-0.0550702	-0.1785677	0.0639879	-0.0606091
50-54	-0.1579634	0.0602180	-0.1599725	-0.1708390	-0.0087575	-0.1726533
55-59	-0.1468544	0.0185544	-0.2365399	-0.1585197	-0.0396098	-0.2213627
60-64	-0.1331373	0.0046522	-0.2617202	-0.1428159	-0.0483801	-0.2256404
65-69	-0.1176974	-0.0019940	-0.2474379	-0.1253526	-0.0535624	-0.2114582
70-74	-0.1008278	-0.0127032	-0.2135741	-0.1073236	-0.0562191	-0.1926942
75-79	-0.0840079	-0.0221242	-0.1760391	-0.0895666	-0.0569043	-0.1709120
80-84	-0.0682132	-0.0287822	-0.1401065	-0.0728106	-0.0548989	-0.1486097
85-89	-0.0537863	-0.0323430	-0.1067072	-0.0574271	-0.0501554	-0.1259542
90-94	-0.0410495	-0.0328545	-0.0768824	-0.0437821	-0.0431549	-0.1033906
95-99	-0.0302452	-0.0307139	-0.0515381	-0.0321648	-0.0347541	-0.0815795
100+	-0.0211379	-0.0250777	-0.0316640	-0.0223087	-0.0241362	-0.0612100
