# Supplementary material for: Modeling Age-Specific Mortality for Countries with Generalized HIV Epidemics
Source: PLoS One. 2014 May 22;9(5):e96447. doi: 10.1371/journal.pone.0096447 (PMC4031074; doi:10.1371/journal.pone.0096447)
Supplement: Table S2 — Coefficients for modeled weights as a function of and prevalence. Given values of and prevalence, these models will produce weights that when inserted into Equation 1 will produce a complete set of age-specific mortality rates. (PDF) [file pone.0096447.s007.pdf]

Modeling Age-Specific Mortality for Countries with Generalized HIV Epidemics: Table S2

Table S2. Coefficients for modeled weights $\omega_{i,\cdot}$ as a function of e_0 and prevalence. Given values of e_0 and prevalence, these models will produce weights $\hat{\omega}_{i,\cdot}$ that when inserted into Equation 1 will produce a complete set of age-specific mortality rates.

Weight	Intercept	e_0	Prevalence
Female: Africa			
$\omega_{1,f,a}$	-13.358	-0.236	—
$\omega_{2,f,a}$	3.004	-0.043	-0.186
$\omega_{3,f,a}$	-1.869	0.036	-0.046
Female: Non-African			
$\omega_{1,f,c}$	-7.348	-0.348	—
$\omega_{2,f,c}$	5.228	-0.078	-0.546
$\omega_{3,f,c}$	-2.323	0.054	-0.166
Male: Africa			
$\omega_{1,m,a}$	-13.559	-0.246	—
$\omega_{2,m,a}$	2.998	-0.046	-0.181
$\omega_{3,m,a}$	-1.713	0.035	-0.050
Male: Non-African			
$\omega_{1,m,c}$	-6.066	-0.393	—
$\omega_{2,m,c}$	5.253	-0.083	-0.569
$\omega_{3,m,c}$	-1.643	0.046	-0.135
