# Supplementary material for: Modeling Age-Specific Mortality for Countries with Generalized HIV Epidemics
Source: PLoS One. 2014 May 22;9(5):e96447. doi: 10.1371/journal.pone.0096447 (PMC4031074; doi:10.1371/journal.pone.0096447)
Supplement: Table S3 — Coefficients for modeled weights as a function of and prevalence. Given values of and prevalence, these models will produce weights that when inserted into Equation 1 will produce a complete set of age-specific mortality rates. (PDF) [file pone.0096447.s008.pdf]

Modeling Age-Specific Mortality for Countries with Generalized HIV Epidemics: Table S3

Table S3. Coefficients for modeled weights $\omega_{i,\cdot}$ as a function of ${}_5q_0$ and prevalence. Given values of ${}_5q_0$ and prevalence, these models will produce weights $\hat{\omega}_{i,\cdot}$ that when inserted into Equation 1 will produce a complete set of age-specific mortality rates.

Weight	Intercept	${}_5q_0$	Prevalence
Female: Africa			
$\omega_{1,\cdot}$	-29.846	25.371	0.091
$\omega_{2,\cdot}$	-0.370	6.715	-0.158
$\omega_{3,\cdot}$	0.550	-3.371	-0.059
Female: Non-African			
$\omega_{1,\cdot}$	-34.380	52.958	—
$\omega_{2,\cdot}$	-0.891	12.352	-0.507
$\omega_{3,\cdot}$	1.981	-9.597	-0.208
Male: Africa			
$\omega_{1,\cdot}$	-30.209	24.613	0.096
$\omega_{2,\cdot}$	-0.399	6.179	-0.158
$\omega_{3,\cdot}$	0.543	-2.993	-0.059
Male: Non-African			
$\omega_{1,\cdot}$	-35.097	53.381	—
$\omega_{2,\cdot}$	-1.031	12.140	-0.510
$\omega_{3,\cdot}$	1.902	-7.459	-0.178
