# Supplementary material for: Modeling Age-Specific Mortality for Countries with Generalized HIV Epidemics
Source: PLoS One. 2014 May 22;9(5):e96447. doi: 10.1371/journal.pone.0096447 (PMC4031074; doi:10.1371/journal.pone.0096447)
Supplement: Table S4 — Coefficients for modeled weights as a function of , , and prevalence. Given values of , , and prevalence, these models will produce predicted weights that when inserted into Equation 1 will produce a complete set of age-specific mortality rates. (PDF) [file pone.0096447.s009.pdf]

Modeling Age-Specific Mortality for Countries with Generalized HIV Epidemics: Table S4

Table S4. Coefficients for modeled weights $\omega_{i,\cdot}$ as a function of ${}_5q_0$, ${}_{45}q_{15}$, and prevalence. Given values of ${}_5q_0$, ${}_{45}q_{15}$, and prevalence, these models will produce predicted weights $\hat{\omega}_{i,\cdot}$ that when inserted into Equation 1 will produce a complete set of age-specific mortality rates.

Weight	Intercept	${}_5q_0$	${}_{45}q_{15}$	Prevalence
Female: Africa				
$\omega_{1,\cdot}$	-31.676	16.418	10.014	—
$\omega_{2,\cdot}$	0.249	10.966	-4.134	-0.108
$\omega_{3,\cdot}$	0.768	-1.874	-1.456	-0.041
Female: Non-African				
$\omega_{1,\cdot}$	-37.580	-4.386	38.441	—
$\omega_{2,\cdot}$	-0.524	22.738	-6.421	-0.423
$\omega_{3,\cdot}$	2.083	-6.703	-1.789	-0.185
Male: Africa				
$\omega_{1,\cdot}$	-32.567	14.655	10.925	—
$\omega_{2,\cdot}$	0.555	10.937	-4.846	-0.107
$\omega_{3,\cdot}$	0.686	-2.278	-0.728	-0.051
Male: Non-African				
$\omega_{1,\cdot}$	-38.564	18.561	25.961	—
$\omega_{2,\cdot}$	-0.573	18.304	-4.250	-0.457
$\omega_{3,\cdot}$	1.976	-6.452	-0.694	-0.170
