# Supplementary material for: Modeling Age-Specific Mortality for Countries with Generalized HIV Epidemics
Source: PLoS One. 2014 May 22;9(5):e96447. doi: 10.1371/journal.pone.0096447 (PMC4031074; doi:10.1371/journal.pone.0096447)
Supplement: Table S5 — Mean Absolute Error for ages 0–75 for all model life table systems by sub-ranges of HIV prevalence. HIV prevalence ranges are shown at the top of each column. All numbers in this table are per 1,000. (PDF) [file pone.0096447.s010.pdf]

Modeling Age-Specific Mortality for Countries with Generalized HIV Epidemics: Table S5

In-sample validation: Mean Absolute Error at varying levels of HIV prevalence

We use the 1% prevalence threshold to select countries for inclusion in this analysis as it is the internationally recognized threshold for a generalized epidemic and is likely the result that most potential users will need, but the model performs well at very low as well as very high levels of HIV prevalence. Of the 320 life tables in the WPP 2010 dataset 142 have HIV prevalence below 1%. We included life tables from earlier periods from countries with generalized epidemics to capture mortality patterns unaffected by HIV-related deaths so the model is able to replicate mortality at very low or even 0% prevalence.

We performed an in-sample validation fitting each of the 320 mortality rate schedules in the WPP 2010 dataset with all three input versions of our model and the four comparison models: the WHO modified logit model, the Coale and Demeny model life tables, the UN model life tables for developing countries, and the Log-Quad model by Wilmoth et al. Table S5 and Figure S5 show the mean absolute error (MAE) among all life tables for ages 0-75 for four sub-ranges of HIV prevalence: less than one percent, one to five percent, five to 20 percent, and greater than 20 percent. Smaller numbers suggest a better fit. The HIV MLT model with all three input combinations performs fairly consistently in terms of the mean absolute error for all life tables with little change in the MAE at various prevalence levels. Compared to the four other models, the HIV MLT model performs better at nearly every prevalence level for both males and females. Although we see an increase in the error for our model as prevalence rates increase, the HIV MLT model fits better than any existing model at these very high prevalence levels and all other models see much larger increases as prevalence rises. These results indicate that our model is able to perform well at all levels of HIV prevalence.

Table S5. Mean Absolute Error for ages 0-75 for all model life table systems by sub-ranges of HIV prevalence. HIV prevalence ranges are shown at the top of each column. All numbers in this table are per 1,000.

	Male				Female			
Model	0-.99	1-4.99	5-19.99	20+	0-.99	1-4.99	5-19.99	20+
HIV MLT								
input: e_0	3.1	3.2	4.5	4.2	2.9	2.9	4.0	4.8
input: ${}_5q_0$	2.9	3.0	3.7	3.6	2.7	2.8	2.9	4.0
input: ${}_5q_0$ and ${}_{45}q_{15}$	2.4	2.9	3.4	3.4	2.5	2.8	3.1	3.7
WHO	5.7	5.1	7.2	10.1	4.2	3.9	5.9	11.3
CD	3.3	3.0	5.9	10.3	1.9	2.7	6.4	13.8
UN	6.0	5.7	7.6	10.8	5.7	4.1	4.3	7.1
Log-Quad	3.2	4.1	7.0	11.9	3.0	3.5	5.7	10.1
